# Modeling protein folding in vivo

**DOI:** 10.1186/s13062-018-0217-6

**Published:** 2018-07-06

**Authors:** Irina Sorokina, Arcady Mushegian

**Affiliations:** 1 Strenic LLC, McLean, VA 22102 USA; 2 McLean, USA

**Keywords:** Protein folding in vivo, Protein folding machine, Co-translational protein folding, Ribosome, Trigger factor, Chaperone, Metastable protein, Fast protein folding, Peptide rotation, Motions at the peptidyl transferase center

## Abstract

**Abstract:**

A half century of studying protein folding in vitro and modeling it in silico has not provided us with a reliable computational method to predict the native conformations of proteins de novo, let alone identify the intermediates on their folding pathways. In this Opinion article, we suggest that the reason for this impasse is the over-reliance on current physical models of protein folding that are based on the assumption that proteins are able to fold spontaneously without assistance. These models arose from studies conducted in vitro on a biased sample of smaller, easier-to-isolate proteins, whose native structures appear to be thermodynamically stable. Meanwhile, the vast empirical data on the majority of larger proteins suggests that once these proteins are completely denatured in vitro, they cannot fold into native conformations without assistance. Moreover, they tend to lose their native conformations spontaneously and irreversibly in vitro, and therefore such conformations must be metastable. We propose a model of protein folding that is based on the notion that the folding of all proteins in the cell is mediated by the actions of the “protein folding machine” that includes the ribosome, various chaperones, and other components involved in co-translational or post-translational formation, maintenance and repair of protein native conformations in vivo. The most important and universal component of the protein folding machine consists of the ribosome in complex with the welcoming committee chaperones. The concerted actions of molecular machinery in the ribosome peptidyl transferase center, in the exit tunnel, and at the surface of the ribosome result in the application of mechanical and other forces to the nascent peptide, reducing its conformational entropy and possibly creating strain in the peptide backbone. The resulting high-energy conformation of the nascent peptide allows it to fold very fast and to overcome high kinetic barriers along the folding pathway. The early folding intermediates in vivo are stabilized by interactions with the ribosome and welcoming committee chaperones and would not be able to exist in vitro in the absence of such cellular components. In vitro experiments that unfold proteins by heat or chemical treatment produce denaturation ensembles that are very different from folding intermediates in vivo and therefore have very limited use in reconstructing the in vivo folding pathways. We conclude that computational modeling of protein folding should deemphasize the notion of unassisted thermodynamically controlled folding, and should focus instead on the step-by-step reverse engineering of the folding process as it actually occurs in vivo.

**Reviewers:**

This article was reviewed by Eugene Koonin and Frank Eisenhaber.

## Current views of protein folding are based on data from a biased sample of proteins

Proteins are the most important and fascinating class of biological molecules. They come in a wide variety of sizes and shapes (reviewed in [1]), perform thousands of functions and possess a wide range of chemical and physical properties. The diversity of their abilities continues to challenge the imagination [2–9]. And yet, all this variety is achieved by folding linear polypeptide chains consisting of only 20 types of building blocks into the specific spatial structures of protein native conformations. The native conformation of each protein arose by natural selection to allow proteins to perform their functions contributing to the organism’s fitness. In vivo, some proteins survive in their active functional forms for hundreds of years [10], whereas others have half-lives of only a few minutes [11]. In vitro, some proteins are capable of retaining their native conformations in extreme conditions, e.g., at temperatures above the boiling point of water [12], whereas others denature, aggregate and lose their activity irreversibly even while kept at the nearly physiological conditions, creating a very costly problem for the pharmaceutical and biotechnology industries [13–16].

All this variability makes the experimental study of proteins in vitro a challenging task. Total chemical synthesis of proteins has been accomplished so far only on a handful of small proteins. The primary source of proteins for in vitro experiments is living cells. To isolate and purify each individual protein from all other proteins in a cell and obtain an active, homogeneous preparation of any protein, experimental protocols must be developed anew; there are no universal purification protocols. The isolation and purification process is always tedious and failure-prone; proteins often lose their activity during the purification, and those that are isolated in an active functional form often do not retain activity for long despite all the efforts, up to the point that the biochemists work in refrigerated cold rooms at 4 C in a quest to slow down protein inactivation by keeping their preparations cold at all times. The specific reasons for the loss of activity during and after the purification process are often difficult to establish, since the isolated proteins can undergo chemical (e.g. oxidation, deamidation) and physical (e.g. denaturation, aggregation) changes. As a result of the difficulty and complexity of protein purification, there is an inherent bias in the sample of proteins that have been available for direct in vitro studies in a homogeneous, soluble, active form; that sample is highly enriched in relatively small, physically and chemically stable molecules. This subset of proteins is what tends to be analyzed in vitro.

Not surprisingly, the representatives of this category of small, stable proteins became the models in the studies of in vitro protein folding and unfolding. The most famous of these studies were the experiments by C.Anfinsen and colleagues, which observed that some small proteins, notably pancreatic ribonuclease (RNAse A), will fold spontaneously to their native conformations from an apparently completely denatured state after the restoration of favorable conditions in vitro; such an ability was postulated – in our opinion, with premature optimism – to be inherent to most proteins. These ideas gave rise to the “thermodynamic hypothesis” stating that “the three-dimensional structure of a native protein in its normal physiological milieu (solvent, pH, ionic strength, presence of other components such as metal ions or prosthetic groups, temperature, and other) is the one in which the Gibbs free energy of the whole system is the lowest” [17]. In other words, under physiological conditions all proteins were assumed to be able to fold spontaneously into their native conformation.

At the time that the thermodynamic hypothesis was gaining acceptance, alternative concepts of kinetically controlled protein folding mechanisms were also widely discussed. The kinetically controlled process can be summarized to say that proteins fold in a guided way along a limited number of kinetically accessible pathways into metastable conformations that are characterized by a local, not global, thermodynamic minimum [18–20]. But the Anfinsen-style unfolding/refolding experiments tipped the scales toward the wider acceptance of the thermodynamic model (reviewed in [21, 22]).

Thus, the most popular theories of protein folding for the last fifty years have rested on the foundation of two assumptions: that protein folding begins from a completely unfolded initial state and that it ends in a native conformation characterized by the minimum of Gibbs free energy. These assumptions also underlie practical applications, such as the development of software for modeling protein folding processes in silico. Further development of these theories gave rise to the model that stated that protein folding occurs through a series of transitions from unfolded conformations through increasingly compact conformations to the native state, and the union of such transitions forms a “folding funnel” [23–25]. To explain how proteins slide down the rugged funnel-shaped energy landscapes without being trapped in local energy minima, a number of additional properties of the energy landscape and of the protein sequences themselves have been postulated, but the assumption that the folding leads to the global minimum of Gibbs free energy remained mostly unchallenged (e.g., [26]). Recently, some models were proposed in which folding landscapes have volcano shapes: in the early steps of the folding process the entropy is reduced due to the formation of some elements of secondary structure, thus the familiar “folding funnel” is placed on a gentle hill [27, 28].

## Why the current models are not satisfactory

Simple and elegant as these models are, they fail to adequately accommodate some common empirical observations. The first one is the widely observed protein physical instability in vitro: most protein preparations that are initially isolated from cells in an active native conformation are not stable in vitro and inevitably denature and lose such native conformation (reviewed in [13–16]). The second is the body of experimental observations that even seemingly stable proteins, once experimentally denatured in vitro in isolation from other cell components, are often unable to fold back into their native conformations upon return to physiological conditions [29–35]. This phenomenon is observed for all classes of proteins, though it becomes more obvious and almost universal for proteins of larger sizes. It has been shown that many such proteins require the assistance of molecular chaperones for successful folding (reviewed in [36]). The current view of the mechanisms of chaperone activity tends to conform to the hypothesis of the funnel-shaped energy landscape; in this account, chaperones are considered to be aids that help the proteins fold by not allowing them to be caught in the non-native kinetic traps [37–40]. The exact mechanism of action of any chaperone system, however, remains unclear.

We are now witnessing the emergence of a third observation that casts doubt on the applicability of the thermodynamic folding model to the majority of proteins: despite the tremendous intellectual and computational efforts invested into modeling of protein folding in silico, software based on the current thermodynamic theory of folding is able to model the folding paths of only very short proteins, and the process is slow [41–43]. In other words, the model in which a polypeptide with a random starting conformation slides down the energy funnel towards the thermodynamic minimum, reducing its free energy at every step in the process, does not appear to yield successful in silico recapitulation of the folding pathways for the majority of proteins.

Some progress has been made in solving the less challenging problem of predicting proteins’ final native structures, but, as evidenced by multiple years of assessing the results of blind prediction of protein structures, the best predictions rely on quantitative approaches of a different kind, namely probabilistic modeling of protein sequence evolution and string-matching algorithms for sequence database searches. The essence of this set of methods is to infer the structure of the target protein from the known, experimentally determined (by X-ray crystallography or nuclear magnetic resonance) structures of its database homologs / templates [44, 45]. It has been noted that physics-based approaches improve such template-predicted protein structures only slightly and mostly in the local regions that do not align well to the homologous sequences [45, 46].

In cases of completely template-free modeling, where no homologs have been structurally characterized, the most powerful approach is also alignment-based: the main method there is to infer spatial contacts in the structure by examining correlated amino acid changes in distant alignment positions, and to predict the fold from the pattern of those contacts. As with sequence-structure modeling, the main signal comes from covariation statistics tested within a molecular evolutionary framework, not from physical potentials [45, 47]. While these sequence similarity-based computational approaches are relatively successful in predicting the native protein structures, they are not equipped to solve a more challenging problem of identifying the folding intermediates, mostly due to the fact that the experimental data about such intermediates is lacking. This state of affairs is unfortunate, because the knowledge of the folding pathways of specific proteins, far from being a purely academic concern, is critical for our understanding of some of the most devastating diseases, such as Alzheimer’s disease and other conditions recognized as protein folding pathologies [48–50]. Software capable of modeling the in vivo folding pathways and accurately predicting folding intermediates of specific proteins would allow us to identify the points of intervention in the pathological folding process. That is why the aforementioned limited success of physics-based software for protein folding is so frustrating.

## We have been modeling the wrong process. It is time to reconsider

In our opinion, the slow progress in modeling the total folding process in silico utilizing the thermodynamics-based modeling approach is not due to the lack of computational power, but to the fact that the wrong process is being modeled. Currently when we try to reconstruct in silico the entire process of protein folding, we model something that is similar to what happens in vitro in those rare instances when select small proteins that are produced in an artificial process of total chemical synthesis fold spontaneously into their native conformations. Proteins obtained by total chemical synthesis presumably start the folding process from random chain conformations. These proteins fold slowly – it takes hours or days to fold the synthesized precursor polypeptide into an active conformation – and the yield of the correctly folded proteins is quite low [51–53]. We can predict that, as we try to produce a variety of larger proteins by chemical synthesis in vitro, we will encounter the complete inability of larger proteins obtained by this method to fold spontaneously, no matter how long we are willing to wait for them to fold. Similarly, attempts to fold large proteins in silico utilizing the current approaches will fail too, no matter how great the computational power we dedicate to it. In contrast, protein molecules in vivo are synthesized and quickly folded into native conformations, regardless of the protein size or amino acid composition, so protein folding mechanisms and pathways in vivo must be different. Therefore in vivo folding processes must be modeled differently: we should take into account the interactions of the folding protein with the cellular components, and accommodate the empirical observations that suggest that native conformations of many proteins are metastable.

## The majority of proteins may be metastable

The latter part of the proposed perspective is supported now by a considerable amount of experimental data. The classic idea that the folding process is kinetically controlled and that the native conformations of proteins may not be at the global minimum of the Gibbs free energy keeps receiving support from the experimental studies of individual proteins [54–59]. In at least one case, it has been already shown experimentally that the native conformation of a protein (the α-lytic protease) has higher Gibbs free energy *ΔG* than its denatured forms [58]. We can safely assume that many more proteins have similar thermodynamic properties. The α-lytic protease has high enough kinetic barrier to persist in a metastable native conformation during the isolation and purification process, thus allowing its experimental study in vitro. Many more proteins that may possess similar thermodynamic properties and not as high kinetic barriers to protect their native conformations have higher chances of unfolding during the purification process and never offer an opportunity to study them in vitro in their active homogeneous form. In fact, it is a very common occurrence in biochemistry and biotechnology practice that protein purification fails due to the denaturation or “misfolding” of a target protein. Unfortunately, the results of such failed experiments are usually considered not worth publishing, so there is no statistical data that would allow us to estimate the percentage of such proteins. Moreover, for the majority of those proteins that were available for studies in vitro, the *ΔG* of folding is estimated to be within −5-15 kcal/mol, meaning that their native conformations are only marginally more stable thermodynamically than their unfolded, inactive conformations [14, 20, 60–63]. This net conformational stability is the result of a delicate balance between large stabilizing enthalpy and large destabilizing entropy contributions, and the resulting *ΔG* of the folding process cannot be measured experimentally. While the enthalpy change of the unfolding/folding process can be determined experimentally by microcalorimetry techniques [64], the entropy change has to be calculated indirectly and, depending on methodology of such calculations, the resulting numbers can differ [65], casting doubts on the accuracy of the available folding *ΔG* values. In other words, the conventionally accepted marginal thermodynamic stability of proteins is just an estimate, and it is a matter of belief that all proteins must be thermodynamically stable, even if barely.

In our opinion, given the diversity of proteins, their functions, and their physical and chemical properties, we should assume that there must exist a similarly diverse continuum of their folding energy landscapes. At one extreme we will find stable proteins, whose native structures have lower Gibbs free energy than their unfolded states. The other extreme may be populated with larger proteins that have a higher Gibbs free energy in their native conformation than in the unfolded state. The vast majority of proteins that fill the continuum between those extremes may be marginally thermodynamically stable or become thermodynamically unstable with fluctuations in the environmental conditions, even within the physiological range. The thermodynamically unstable proteins can still retain their native conformations in the metastable state, protected by kinetic barriers.

Why is it that such a possibility is rarely considered? After all, if a protein is kept in the native conformation in a transient metastable state by a kinetic barrier, it should not matter whether the *ΔG* of folding is a small negative or a small positive number – from the point of view of a living cell, it is only important that the energy barrier keeping the protein folded is high enough to allow protein to stay in that active conformation for the duration of its useful life in vivo. Such scenario, however, would demand an explanation: how did the protein molecule end up folded in the first place into a native conformation that has a higher free energy than the unfolded molecule? Such a state would be impossible to achieve in vitro in the absence of an accessible external source of energy. But it is possible in a living cell – a system that is neither closed nor at equilibrium.

## How to fold thermodynamically unstable proteins

Thus, if we want to build a more universal physical model of protein folding in vivo, applicable to proteins of all sizes with various thermodynamic properties, we must postulate the existence of a protein folding machine. Such a postulate would allow us to build a physical model of folding that would more realistically describe the folding processes actually taking place in a living cell rather than in vitro and start closing the great gap that exists between studies of protein folding in vivo and in vitro [66].

The postulated protein folding machine should be able to apply forces to the polypeptide chain and utilize external energy sources to force the peptide into a higher energy state whenever necessary during the folding process, thus allowing the polypeptide to overcome high kinetic barriers during folding and relax into a final native conformation that may or may not be in a higher free energy state than the unfolded ones. To identify candidate components of this folding machine, we should examine any cellular entity, factor, or subsystem that could use an external source of energy to create an environment to facilitate the folding process or apply any force to a folding peptide. Some parts of the folding machine are easily recognized as such, for example chaperone systems; others might not be immediately obvious.

In a recent attempt to identify the components of the protein folding machine, we described a possible co-translational twisting of the nascent peptides by a ribosome working in conjunction with the ribosome-associated chaperone complex [67, 68]; these chaperones located at the exit of the ribosome tunnel have been collectively named “nascent chain welcoming committee” [69, 70]. Initially, we were intrigued by the possibility that some mechanical forces could be applied to peptides in vivo, compacting the peptide backbone into conformations with reduced entropy, thus allowing the protein to climb up the energy hill in the early stages of folding, or, possibly, forcing the peptide into some strained conformations with higher potential energy, from which the peptide would then relax into otherwise kinetically unaccessible states. We explored different types of mechanical perturbations that could conceivably happen to the polypeptide in the living cell: the protein backbone might be pushed, pulled, stretched, bent, rotated and twisted, or otherwise manipulated to facilitate the folding process. Out of all examined possibilities, the twisting of the backbone was the most promising one. Twisting has been already studied in the context of higher order structure of biopolymers, e.g. DNA. If a torque force is applied to a linear biopolymer, and its mobility is restricted at a distal point at the same time, the resulting tension induces turns, twists, coils and other secondary structures in the molecule, as is well known in the case of supercoiled double-strand DNA but also applies to single-strand DNA regions when their ends are not free to move [71–73]. The protein backbone is often modeled as a freely-jointed chain due to its ability to rotate around N-Cα and Cα-C bonds in every monomer. If, however, the backbone rotation is forced to occur only in one direction, as would be the case with consistent application of the torque force, eventually some dihedral angles phi and psi would be pushed into sterically disallowed positions, which are described in Ramachandran plots [74, 75], limiting the rotation in those positions and making possible the accumulation of tension in the peptide backbone. An example of such possible local torsion-induced strain in the native structure of bacterial methyl transferase has been described [76].

**Fig. 1:**
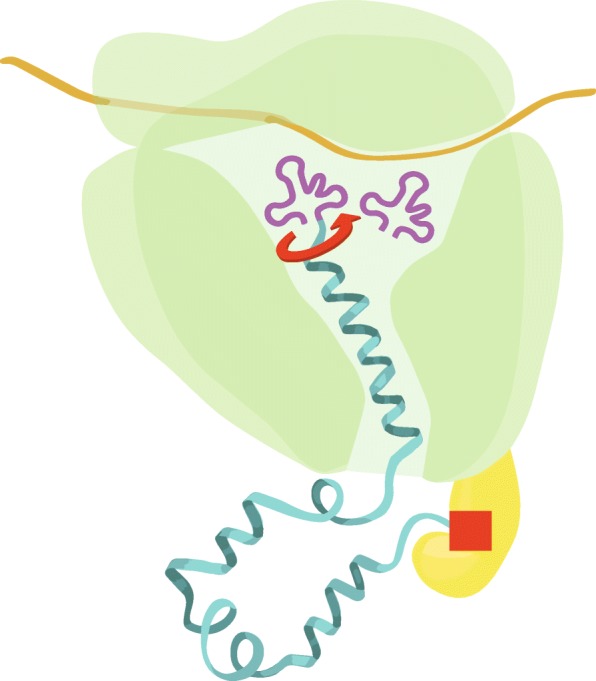
Ribosome/chaperone complex as a protein folding machine capable of manipulating the polypeptide backbone. Red arrow shows rotation of the C-terminus of the nascent peptide in the peptidyl transferase center. Red square indicates the movement restriction and stabilization of the N-terminus by trigger factor or other welcoming-committee anchor proteins

By searching the literature, we have found that the protein backbone twisting hypothesis may indeed be supported by published experimental data. The ribosome may be able to rotate the C-terminus of the nascent polypeptides at its peptidyl transferase center, where the 3′ end of peptidylated tRNA undergoes an otherwise unexplained 180-degree rotation upon translocation from the A-site to P-site [77] while the N-terminal regions of the nascent peptides may be rotationally restricted first by occlusions in the ribosome exit tunnel [78], and next by steric capture mediated by the ribosome welcoming committee chaperones, most importantly trigger factor in bacteria and the nascent polypeptide-associated complex in archaea and eukaryotes [79–81]. Figure 1 schematically illustrates two main components of the ribosome/chaperone mechanism: rotation of the C terminus of the nascent peptide and mobility restriction of the N-terminus. Such motions may facilitate the formation of the early elements of the nascent peptide’s secondary structure, most obviously alpha helices, but also possibly, depending on the specific local amino acid sequence, bends, loops and other structural elements. The idea that the protein folding occurs co-translationally is, of course, not new (for reviews, see [82–84]), but previous attempts to elucidate the mechanism of co-translational folding, as far as we know, did not take into consideration the mechanical or other forces that the ribosome may apply to the nascent peptide. We suggest that the forces applied to the nascent peptide manipulate it into an unstable high-energy conformation that is then stabilized by the interactions first with the elements of the ribosomal tunnel and then with the welcoming committee chaperones. These steps occur before the start of the formation of tertiary structure and of the emergence of enthalpy-reducing long-distance interactions. We emphasize that the main role of the welcoming committee chaperones is to stabilize the peptide in the high-energy state until the formation of the tertiary structure starts.

The analysis of energy expenditure in the process of protein biosynthesis suggests that the energy needs of peptidyl transferase reaction are fully supported by the ester bond energy of aminoacyl-tRNA, which is formed by an ATP-dependent reaction outside of the ribosome. In addition to this, the ribosome expends about 15 kcal of excess energy per each newly added amino acid, as the ribosome-associated translation factors hydrolyze two GTP molecules in each elongation cycle ([85], pp. 55–56; [86]). This significant excess of energy does not appear to be coupled with any biosynthetic or regulatory process, and has been thought mostly to dissipate as heat. A.S.Spirin noted that “The meaning of the release of such a tremendous excess of energy is an enigma and extremely interesting problem in molecular biology” ([85], p. 57). A possibility has been brought up that part of this energy is spent to reduce the entropy of the nascent chain due to the ordered arrangement of the amino acids along the chain of the synthesized protein (ibid). This reduction of conformational entropy of the nascent peptide would indeed be the first contribution toward raising its Gibbs free energy. GTP hydrolysis by the translation factors appears also to be needed for improving translation fidelity and for promoting ribosome structural rearrangements (reviewed in [87]), but the energy requirements of these processes are not known. We hypothesize that some of the remaining excess of energy might be partially transfered to the nascent peptide in other forms, like the torque-induced strain described above.

To summarize, analyses of energy balance during protein synthesis suggest that at the very least, the Gibbs free energy of the peptide at the early stages of folding is elevated due to the reduced conformational entropy. Speculating further, we can allow the possibility that either the whole polypeptide chain or parts of it at some point in the process additionally gain higher potential energy due to the torsional strain. Either way, the mechanical forces that the ribosome-welcoming committee complex applies to the nascent peptide are able to “wind up” the peptide into a high-energy state, which would then make possible a quick collapse into the tertiary structure that is stabilized by the formation of the hydrophobic and other enthalpy-reducing native contacts. It is during the final collapse into the native conformation that the remaining excess energy would dissipate as heat. This proposed sequence of events during co-translational folding would explain how proteins fold so quickly in vivo. Other mechanisms by which a higher energy state of the nascent peptide is induced and maintained by the interaction with the ribosomal components should also be explored, even if the proposed mechanical twisting of the polypeptide is experimentally proven to be incorrect. Experimental studies of other mechanical forces applied to the nascent peptide by the ribosome have already begun [88–91].

The ribosome-welcoming committee complex is likely to be only a part of a much more intricate cellular protein folding machine. Other parts of the machine may include the signal recognition particle, the translocon, specialized protein secretion systems, and networks of diverse classes of chaperones active in various cellular compartments. Much like the ribosome and its associated factors, the majority of these complexes can simultaneously be protein-anchoring devices and energy sources, and much like in the case of the ribosome, their energy balances in many cases are currently not well understood (see, e.g., [92]). These complexes should be considered as additional modules of the protein folding machine, possibly enabling co-translational or post-translational energy boosts for the formation, maintenance or repair of the protein native conformations in vivo. The forces that they apply to the proteins must be examined to determine if and how they may serve these roles. For example, the chaperone complex GroEL-GroES is currently viewed as an entity that creates a “sanctuary” for a partially unfolded protein allowing the polypeptide to fold itself back to the native state while preventing its aggregation with other unfolded proteins (e.g., [93, 94]). If we view the GroEL-GroES complex as part of the folding machine, we may want to study whether it plays a much more active role, e.g. whether it might squeeze water molecules out of a partially denatured protein. This possibility seems plausible in light of recent experiments showing that unfolding starts with the protein structure becoming less tight, thus allowing water to penetrate [95]. Along these lines, it has been recently proposed that ATP hydrolysis by GroEL-GroES may be directly coupled with the non-equilibrium stabilization of protein conformations [96].

## Imagining folding energy landscapes in vivo

A complete accounting of the mechanical or other forces exerted by the ribosome, chaperones and other parts of the folding machine on the polypeptide, and the determination of the energy balance for each of those interactions, will be essential for the quantitative modeling of the co-translational and post-translational folding of individual proteins in vivo. In the meantime, we propose a general view of the energy landscapes that is relevant to protein folding in vivo, takes into account the existence of the protein folding machine, and agrees well with the empirical observations of pervasive protein physical instability, inability of large proteins to refold in vitro, and fast folding of the same proteins in vivo.

**Fig. 2:**
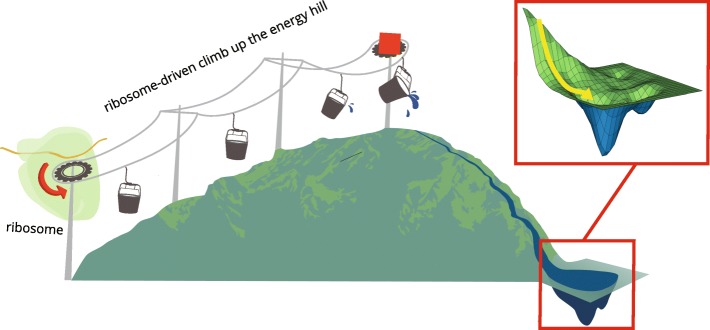
The protein folding energy landscape of a small protein that is thermodynamically stable in its native conformation. The fast folding pathway in vivo starts from an unstable high-energy state at the top of the mountain. The entropy of the nascent peptide is reduced and the backbone conformational strain is introduced by the motions of the ribosome/chaperone complex in the process that is described in [67, 68] and in the text. Yellow arrow shows fast folding pathway in vivo

**Fig. 3:**
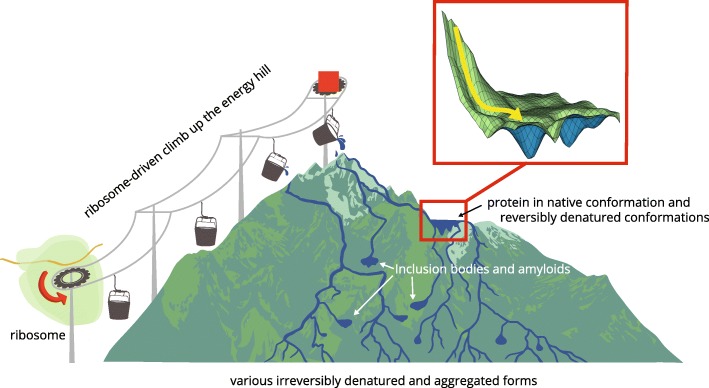
The protein folding landscape of a protein that is metastable in its native conformation. The native fold of this protein occupies a metastable local free energy minimum shown as a mountainside lake, and it is higher than the free energy of the majority of its denatured conformations. This protein can only be folded in vivo, and the folding must start from a higher energy state, at the top of the mountain. The entropy of the nascent peptide is reduced and the backbone conformational strain is introduced by the motions of the ribosome/chaperone complex in the process that is described in [67, 68] and in the text. Yellow arrow shows fast folding pathway in vivo. Many of the denatured forms of this protein have lower Gibbs free energy than the native conformation, and their spontaneous unassisted refolding is not possible. Reversibly and irreversibly denatured forms of the protein occupy multiple positions down the mountain slope

As already discussed, there must exist a variety of folding landscapes that matches the variety of thermodynamic properties of native states of different proteins. Figures 2 and 3 show two of the possible landscapes. Considering that the debates about the exact physical meaning of the “folding energy landscape” and the complexity of its shape are ongoing [97, 98], we emphasize that the energy landscapes that are depicted in Figs. 2 and 3 are provided as mere illustrative schematic representations of parts of the energy landscapes that are relevant to the folding process. Our aim here is to describe some very general features of such landscapes, focusing on their compatibility with the existing experimental data.

Figure 2 illustrates a folding landscape applicable to a small and thermodynamically stable protein that is capable of folding both in vitro and in vivo. It incorporates the familiar funnel-shaped feature that is relevant to folding in vitro from a completely unfolded state, with a brim of the funnel occupied by various completely unfolded conformations that have higher Gibbs free energy values than the folded native conformation. There is one crucial addition to this traditional shape, introduced by the notion of the protein folding machine: the funnel is modified by a distinct folding path that enters the landscape from a much higher energy level. Unlike the random folding trajectories that start at multiple locations throughout the brim of the conventional funnel, this folding path, which is accessible only in vivo, starts with a specific early conformation created co-translationally on a ribosome and proceeds in a channeled way, reaching the native state faster and with better yield than would be possible in the absence of the folding machine. Folding from the completely unfolded conformations most likely never occurs in vivo. Even RNAse A probably follows a different pathway while being folded co-translationally compared to its refolding in vitro.

The high-energy part of the landscape is shown as a mountain, with a “lift” representing the co-translational interactions with the ribosome and welcoming committee chaperones that convey the nascent peptide to a high-energy state. The shape of the mountain must be very rugged; number, dimensions and shapes of its ridges and ravines are determined by the amino acid sequence of the polypeptide and its interactions with the parts of the ribosome and chaperones in the early stages of the co-translational folding. The polypeptide elongation occurs simultaneously with multiple rearrangements of its backbone conformations: the strained local conformations arise in the ribosomal tunnel, relax into various elements of secondary structure, and new local tensions are being created by the ribosome. We deliberately simplified the shape of the mountain since we wanted to illustrate a different feature of this landscape – the steepness of the energy gradient during the co-translational folding. As described earlier, each elongation cycle of protein synthesis by the ribosome generates an estimated excess of ~ 15 kcal from GTP hydrolysis, resulting in ~ 3000 kcal of extra energy per a 200 amino acid protein [85]. We do not know how much of this excess energy is actually spent on the conformational ordering and “winding up” of the nascent peptide, but it is likely to dwarf the depth of the funnel, which is estimated to be only −5-15 kcal/mol for most proteins [61–63].

The other possible category of the folding landscapes is shown in Fig. 3. This is a more complicated landscape that corresponds to the folding of a large protein in vivo and conforms to the experimental data on physical properties of the majority of large proteins observed in vitro. The scenario illustrated here deals with a protein that has a thermodynamically unstable native conformation. The native fold occupies a metastable local free energy minimum depicted as a mountain-side lake, and it is higher than the free energy of the majority of its denatured conformations. The location of this lake on the slope could be higher or lower, depending on the position of the protein on the spectrum from thermodynamically unstable to thermodynamically stable proteins. This large protein can only be folded in vivo, and the folding must start from a higher energy state, which is generated by the ribosome and other parts of the folding machine. Once this large protein is completely denatured, its spontaneous refolding is not possible. Refolding from some partially denatured states might be possible with the assistance of the energy-dependent chaperones such as GroEL-GroES, which could be illustrated by adding more “lifts” on the slopes of this mountain. To account for multiple processes of protein folding, partial denaturation and repair that happen in the cell we would need a much more complicated illustration, with multiple channels and lifts; these complications are deliberately left out of the figure.

**Fig. 4:**
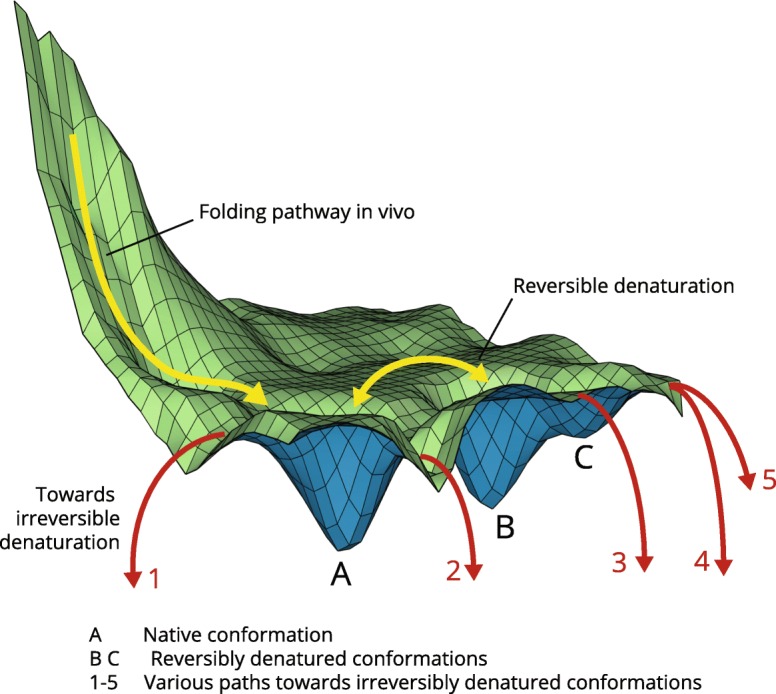
The folding and denaturation pathways of a protein that is metastable in its native conformation. The fast folding pathway in vivo starts from a high-energy state generated by ribosome/chaperone complex in the process that is described in [67, 68] and in the text. The native structure of this protein occupies a metastable local thermodynamic minimum (**A**). If the protein escapes this local thermodynamic minimum, it can either move to another local minimum (**B**, **C**) from which refolding is possible (reversible denaturation), or it can move into conformations with much lower Gibbs free energy, from which it cannot refold (irreversible denaturation). Multiple red arrows illustrate the multiplicity of the denaturation pathways

In a simplified form, the folding of this large protein is characterized by the existence of one fast and efficient co-translational folding pathway and multiple denaturation pathways, as shown in Fig. 4. The native structure of this protein occupies a metastable local thermodynamic minimum (position A on Fig. 4). If the protein escapes this local minimum, two major possibilities exist – it can move to another local minimum (positions B and C), or it can slide much lower down the mountainside into conformations that have much lower Gibbs free energy. These two outcomes would correspond, respectively, to empirically observed reversible and irreversible denaturation of the same protein. Biochemists have recognized and described two different types of denaturation long ago [29]. The mechanisms of irreversible denaturation are still incompletely understood and are under debate. Irreversible denaturation is often attributed to chemical modification or aggregation of proteins. However, in detailed studies of irreversible denaturation accompanied by deamidation and aggregation, it has been established that irreversible denaturation happens first, and both deamidation and aggregation occur much later and therefore cannot be the cause of irreversible denaturation (e.g., [31, 32]). Irreversible denaturation could be also due to the formation of an altered conformation with a high kinetic barrier to refolding [33].

Our model offers an explanation of the irreversible denaturation that does not depend on chemical modification or aggregation of proteins: irreversible denaturation of some proteins may be the result of their completely unfolded conformations having lower Gibbs free energy than their native conformation. In this view, reversible denaturation of such protein is possible when the protein is only partially unfolded and retains some essential parts of the original structure. The Gibbs free energy of such denaturation ensemble is higher than the Gibbs free energy of the native structure, as shown in positions A, B and C in Fig. 4. There is ample evidence that denatured proteins indeed often contain significant amount of residual local structure [99–104]. When such protein is subjected to harsher denaturing conditions and the remnants of the native conformation are lost, the Gibbs free energy of the unfolded forms becomes lower than that of the native conformation, and the unassisted return to the native conformation becomes impossible, the denaturation becomes irreversible. The multiple paths down the energy mountain toward irreversibly denatured states are shown by arrows 1–5 on Fig. 4.

The multiplicity of the unfolding paths in our model is based on the experimental data suggesting that the unfolding proceeds differently depending on the type of denaturing agent: heat, chemical, pressure and force-induced denaturation processes result in different unfolded conformational ensembles [105]; for example, it has been shown that the backbone dihedral angle distributions for a protein unfolded under force and one unfolded by chemical denaturant are very different [106]. The activation energies of the unfolding must also vary depending on the type of the denaturant.

## Conclusion and further directions

To summarize, for the majority of proteins there are multiple ways to destroy their native conformation and most likely only a small number of fast and precise ways to produce it; these fast folding pathways are only accessible in vivo with the involvement of the protein folding machine. The early high-energy folding intermediates in vivo are created and stabilized by interactions with the ribosome and welcoming committee chaperones; these conformations would not be able to persist in vitro in the absence of the cellular components. Therefore, if we want to understand how all proteins are being folded in vivo, we need to deemphasize the in vitro bulk denaturation experiments, because none of the brute force denaturation techniques that we are using, whether it is heating, chemical denaturation, applying pressure, etc., are able to reverse the steps of the co-translational folding. It would not make sense to study the making of an intricate jewelry artifact by pounding it with a hammer or melting it in a kiln, nor to learn how to fold an origami flower by soaking it in acid solution; why should we continue doing this with proteins?

The reason why biochemists have been performing bulk protein denaturation-renaturation experiments in vitro is at least understandable, as until recently we were not able to exert precisely controlled forces on single molecules. It is only recently that the single molecule techniques have been approaching the level of sophistication needed for the task, and there is a great hope that soon it will become possible to apply specific kinds of mechanical and other forces to carefully unfold and refold single protein molecules, in ways that would elucidate the in vivo folding pathways.

Meanwhile, in our computational experiments, there is no good reason to limit ourselves to simulating protein unfolding by heat, for example. Instead, in silico unfolding of the known protein structures should be designed as a step-by-step reverse engineering of the co-translational folding. Along the same lines, when we try to computationally model protein folding as it occurs in vivo, we need to take into account the information from the experimental data on the functioning of the peptidyl transferase center of the ribosome and attempt to recreate the secondary structures of the nascent peptide that arise early during the co-translational folding. We must allow the possibility that polypeptide starts folding from a high-energy state and crosses high energy barriers during folding. We have to examine the mechanical forces that might be applied to the backbone of the protein and compare the structures that arise as a result of such in silico experiments with the known protein native structures.

To conclude, we will have a chance of solving the protein folding problem, gain a hope of intervening in the protein folding pathologies, and improve our ability to manufacture artificial proteins with novel properties, only if we learn the principles of operation of the protein folding machine.

## Reviewers’ comments

### Reviewer’s report 1: Eugene V. Koonin, National Center for biotechnology information, National Library of medicine, National Institutes of Health, USA

## Reviewer comments

In this interesting and well-written Opinion article, Sorokina and Mushegian challenge the common wisdom on protein folding based on the Anfinsen postulate and propound a paradigm shift whereby a cellular “folding machine” is essential for the proper folding of all proteins. The nature of the folding machine can be described only in rather general terms by postulating that it consists of the ribosome and a set of molecular chaperones. The authors further propose that the folding machine generates high-energy intermediates that then relax into local free energy minima.

My general impression of this article is quite peculiar. On the one hand, there is not much new here: after all, it is quite obvious that real proteins do not fold in isolation in test tubes, but rather under conditions of molecular crowding within living cells, typically aided by chaperones, and often, co-translationally. But, on the other hand, all the main claims made by the authors are novel and rather shocking as they defy the firmly entrenched beliefs in the protein-folding field. These beliefs, as well explained in the article, essentially boil down to the concept of a folding protein gradually sliding down the folding funnel towards the global free energy minimum. Chaperones are not thought to be fundamental to the folding process, their function being largely to prevent proteins from being trapped in local minima. Sorokina and Mushegian dispense with all these principles, postulating instead that native conformations are local not global minima; the trajectories on the folding landscape are not smooth but rather involve traverse of “high-energy” intermediate states; and that chaperones are essential for folding of all proteins.

Authors’ response: *We are grateful to Dr. Koonin for his support of our work and for a clear summary of our main points.*

## Reviewer comments (continued)

To this reviewer, the concept of Sorokina and Mushegian indeed makes more sense than the current common wisdom in protein folding field. It has to be admitted, however, that the new concept is only making its baby steps and at present, is quite general rather vague. A lot of theoretical and experimental effort is obviously required to put it on a firm ground.

Authors’ response: *We agree.*

## Reviewer comments (continued)

I do not see a need of any substantial modifications to the article, under the crucial understanding that this is presentation of a concept not even a fully developed hypothesis. A couple of terminological issues that can be easily corrected are listed under Minor issues.

Minor Issues.

I am not sure that “welcoming committee of chaperones” is a good phrase. It reads strange to me, I would think of a less extravagant terminology.

Authors’ response: *We agree that the term “welcoming committee” is a bit vague and does not describe the exact role that these proteins play in the folding of the nascent peptides, but this term already has a history of usage in the community that studies protein folding* in vivo*. As far as we know, it was coined in 1989 by Hartley and Helenius* [107]*, in the context of protein folding on the endoplasmic reticulum, and was applied to the ribosome-associated chaperones in a high-profile review in 2000* [108]*.*

## Reviewer comments (continued)

“High energy” intermediates are an important part of the authors’ concept. I suggest being somewhat more careful here. “High energy” pertains to high deltaG, i.e. unstable conformations - perhaps, just say so? In any case, “high energy” has some unnecessary connotations, I would change it.

Authors’ response: *The “high energy” conformations that we discuss are indeed unstable folding intermediates that have Gibbs free energy values higher than either native protein conformation or completely denatured conformations. We added “unstable” in several places for clarity.*

### Reviewer’s report 2: Frank Eisenhaber, Bioinformatics Institute, A*STAR, Singapore

## Reviewer comments

The article is quite philosophical at this point; yet, it raises a few important points that the community should notice.

Sorokina & Mushegian provide a thoughtful compilation of references that report observations that do not really fit into current models of protein folding. They try to come up with a framework of alternative hypotheses that might become the beginning of a new theory of protein folding.

Authors’ response: *We thank Dr. Eisenhaber for a positive assessment of our work.*

## Reviewer comments (continued)

At this stage, there are many points that might draw criticisms. I will detail only a few:

1. The computational folding models operating with physically meaningful interaction potentials between atoms and atomic groups suffer from considerable inaccuracies that might easily be above the distance between folded and unfolded states depending on the energy components included. This matter is not considered in the manuscript at all. So, the lowest free energy approach has never been properly implemented and the calculations have always delivered more or less false models due to this obstacle alone. Of course, if we talk about folding towards conformations with elevated energies, this computing of ensembles near the lowest free energy makes even less sense.

Authors’ response: *Yes, we fully agree that the interaction potentials at this stage may be inaccurate. But we wanted to focus on a much more serious problem that the prevailing folding models are not applicable to the folding processes as they actually occur in the cell. We can not stress enough that the slow progress in modeling the total folding process* in silico *is not due to the lack of computational power or inaccuracies of the force fields, but to the fact that the wrong process is being modeled. When we start studying and modeling what is actually going on when protein is folded* in vivo*, the better accuracy of interaction potentials will follow.*

## Reviewer comments (continued)

2) Many, sometimes repeating phrases in text hide that we do not know much about the actual in vivo participants of folding. The alternative model is expressed in schemes and general formulations, there is little mechanistic substance that can serve as the beginning of a model calculation or of an idea for an experiment. Therefore, the practical implications will remain very limited.

Authors’ response: *The current paper is a follow-up to our previous publications* [67, 68]*, in which we offered a mechanistic model that is based on the rotation of the C-terminus of the nascent peptide by the peptidyl transferase center of the ribosome coupled with the restriction of the rotational mobility of the N-terminus by the welcoming committee chaperones. In* [68] *we have discussed the experimental data suggesting that peptidyl transferase center, ribosome exit tunnel, bacterial trigger factor, its archaeo/eukaryotic analog NAC, and several other members of the “welcoming committee” all participate in the folding process. More direct experimental studies of some of these mechanisms are underway, in the labs of our collaborators and elsewhere.*

## Reviewer comments (continued)

3) For example, the authors repeatedly talk about the “welcoming chaperones” without providing much information what this might physically mean. As it appears to the reviewer, the notion of a “chaperone” is quite weak anyhow, without too much mechanistic explanation, although there is a huge literature about it. It would be good if specific chaperone systems had been reviewed in more molecular detail and how the available insight supports the rather generally formulated views on folding in this work.

Authors’ response: *Please see our response to point 2) above. In our previous publications* [67, 68] *we suggested a molecular mechanism of active protein folding* in vivo*, the likely participating cellular components and their possible roles. The main focus of this paper, however, is different: we want to discuss not the actual mechanics of the cellular protein folding machine but the empirical evidence suggesting that such machine must exist. In the numerous discussions with the colleagues since the two previous publications, we have repeatedly encountered the same question: “Why even consider the possibility that the proteins are being folded by the cellular machinery? Isn't it a common knowledge that assisted folding is not necessary – proteins are able to fold by themselves as shown by Anfinsen and others?”. Thus the main motivation of this paper is to compel the scientific community to investigate the alternative: the majority of proteins are folded by the cellular machinery and are not able to fold spontaneously.*

## Abbreviations

ATP: Adenosine triphosphate
GTP: Guanosine triphosphate
